# Efficient Separation of C-Tetramethylcalix[4]resorcinarene Conformers by Means of Reversed-Phase Solid-Phase Extraction

**DOI:** 10.1021/acsomega.2c03218

**Published:** 2022-12-21

**Authors:** Héctor Manuel Pineda-Castañeda, Mauricio Maldonado, Zuly Jenny Rivera-Monroy

**Affiliations:** Chemistry Department, Universidad Nacional de Colombia, Carrera 45 No 26-85, Building 451, Office 134, Bogotá11321, Colombia

## Abstract

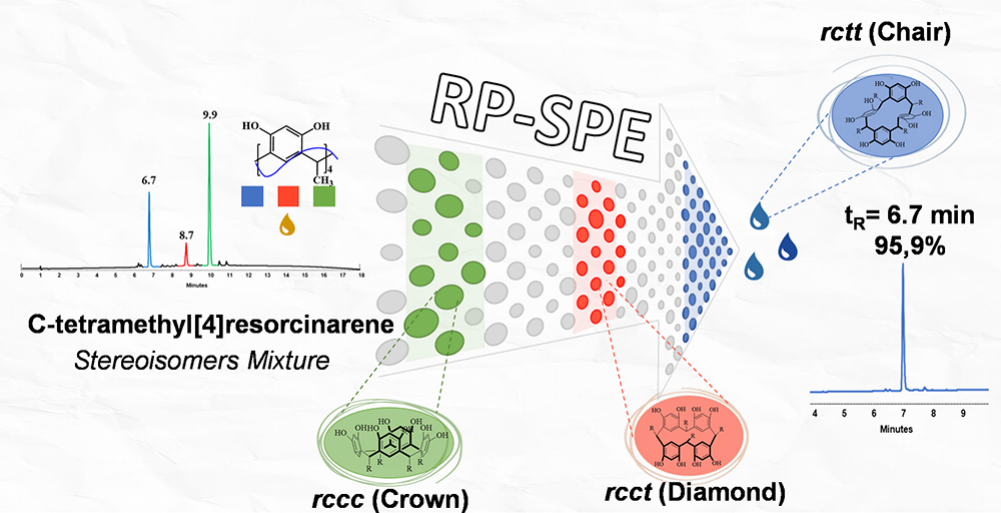

A reversed-phase high-performance liquid chromatography (RP-HPLC) method was developed to study the conformer formation generated during the reaction for obtaining *C*-tetramethylcalix[4]resorcinarene. The chromatographic method was used to design a strategy for purifying the reaction products, using solid-phase extraction columns (RP-SPE) and gradient elution. The chromatographic profiles of the cyclocondensation reaction between resorcinol and acetaldehyde show the presence of three products under the different reaction and precipitation conditions studied. Using RP-SPE, it was possible to enrich the products, which were later characterized by means of RP-HPLC and ^1^H nuclear magnetic resonance (NMR). This investigation explored and established a new method for RP-HPLC analysis and RP-SPE separation of conformational isomers obtained in the formation reaction of *C*-tetramethylcalix[4]resorcinarene.

## 1 Introduction

**Figure 1:**
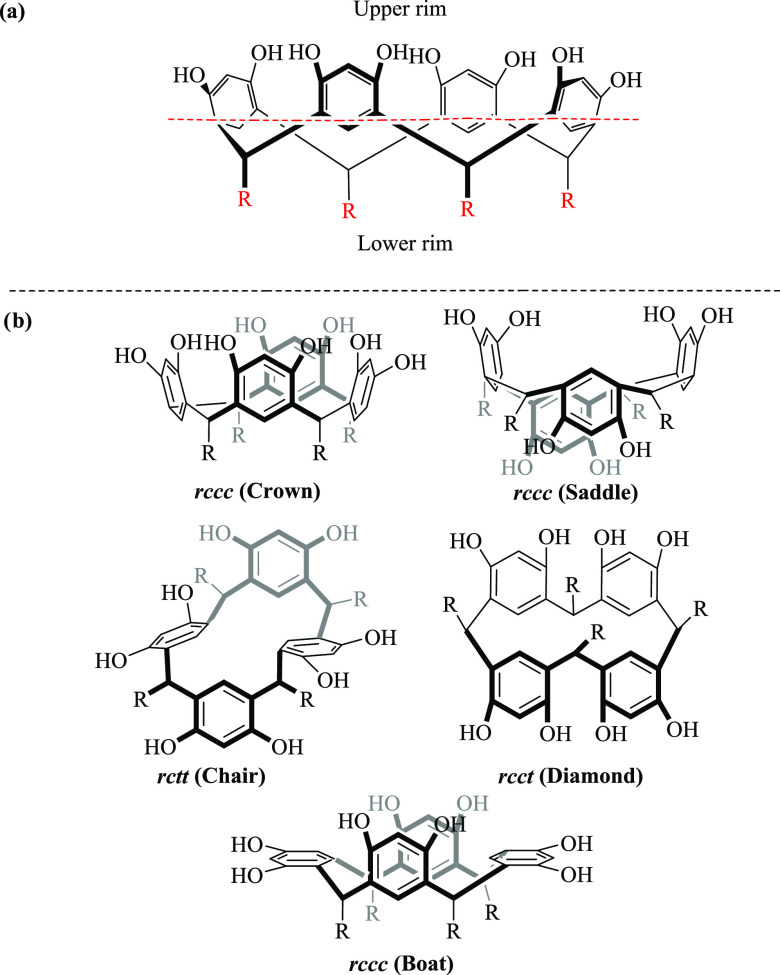
(a) Illustration of the structure of calix[4]resorcinarene crown conformer (upper ring and lower ring). (b) Reported conformational isomers and relative configuration of the substituents on the methylene bridge.

Currently, the synthesis and characterization of macrocycles have generated great interest since they have exhibited great versatility in their use in supramolecular chemistry, catalysis, and separation science, and they have also attracted much interest due to their potential use in designing drug delivery systems.^1−5^ Many receptors and host molecules adopt specific geometries and conformations.^6^ Among these compounds are resorcinarenes, also known as calix[4]resorcinarenes. These are polyhydroxylated macrocyclic compounds derived from resorcinol, first synthesized by Baeyer et al. from aliphatic and aromatic aldehydes.^7,8^ They are made up of four resorcinol rings joined by a bridging atom, usually carbon within a methylene group at positions 4 and 6, producing the formation of a cyclic structure typically represented as a truncated cone with an upper and lower edge (Figure 1a). These bridging atoms are often replaced by aliphatic and/or aromatic chains, allowing the formation of conformational isomers (stereoisomerism).^9^

The versatility of these host systems stems from easy synthetic modifications to either the upper or lower edge of these macrocycles. However, it is known that the resorcinarene conformations of the nucleus (unsubstituted) are quite easily modulated by the reaction conditions when the host is synthesized.^6^ Five possible conformers have been reported: (i) crown, (ii) boat, (iii) saddle, (iv) chair, and (v) diamond (Figure 1b).^10^ Each of these conformations is possible according to aspects such as the position of the resorcinol units and the substituents on the methylene bridges. For most of the cases reported in the synthesis of *C*-tetramethylcalix[4]resorcinarene, the most stable conformation is the crown type. It is established from the deep cavities formed and their stabilization by means of hydrogen bonds mediated by the hydroxyl groups present.^11−14^ Other unusual conformations have been reported, such as *rcct*-diamond for *C*-tetramethylcalix[4]resorcinarene, which is rarely observed,^15^ and the *rcct*-boat for *C*-tetramethyl-2-nitrocalix[4]resorcinarene.^16^ In the C4*v* conformation, intramolecular hydrogen bonds between adjacent phenolic hydroxyl groups preserve the crown structure.

The interconversion of conformational isomers has been reported from the synthesis of a triangular structural brick-wall framework based on *C*-tetramethylcalix[4]resorcinarene and 1,4-bis(pyridyl)ethylene (bpe) formed by converting a polymeric structure with a bowl-to-boat conformational change.^17^ When the conformation is converted from bowl to boat, the intramolecular hydrogen bonds within the upper edge break and change their orientation to the axial direction. This allows hydrogen bonding with the bpe dimers that are redirected into the cavity to facilitate bond formation.^18^ However, sometimes under different synthesis conditions, the presence of conformer mixtures is reported, which makes it difficult to properly characterize each of the products, and there are frequent difficulties for separation since the reaction product behaves as a single compound viewed through TLC and liquid chromatography (LC), but the ^1^H NMR and ^13^C NMR spectrum is consistent with there being two isomers. Alternatives have been sought on this subject, trying to recrystallize the material, but they did not change the relationship of the signals or enrich the conformers.^19^ In an effort to solve these difficulties, methods for separating mixtures of conformational isomers for *C*-tetra(*p*-hydroxyphenyl)calix[4]resorcinarene have been described, where through RP-HPLC two well-resolved signals corresponding to the crown and chair isomers were found, and through the application of an RP-SPE protocol, the separation of the two stereoisomers with high purity and their subsequent characterization using techniques such as FT-IR, ^1^H NMR, and ^13^C NMR were achieved, which confirmed their chemical identity.^20,21^ Although the derivatives of calix[4]resorcinarene are molecules that are of great interest in the chemical and pharmaceutical field given their wide range of applications, there is a limitation in their purification stages. To solve this difficulty, in the present investigation, a new method for RP-HPLC analysis and RP-SPE separation of conformational isomers obtained in the formation reaction of *C*-tetramethylcalix[4]resorcinarene were explored and established.

## 2 Materials and Methods

### 2.1 General Method

^1^H spectra were recorded at 400 MHz on a Bruker Advance 400 instrument. Molar mass was determined with an Agilent 6470 triple quadrupole mass spectrometer. RP-HPLC analyses were performed on a Chromolith RP-18e column (Merck, Kenilworth, NJ, 50 mm), using an Agilent 1200 Liquid Chromatograph (Agilent, Omaha, NE). All products were analyzed on a Bruker Impact II LC Q-TOF MS equipped with electrospray ionization (ESI) in positive mode.

### 2.2 Synthesis of *C*-Tetramethylcalix[4]resorcinarene (Stereoisomers Mixture)

We followed the method reported by Hoegberg et al.^22^ A 1,3-dihydroxybenzene solution (1 mmol) and acetaldehyde (1 mmol) in water (4.0 mL) was added drop by drop to hydrochloric acid (1.0 mL) and was heated at reflux with constant stirring for 1 h. A precipitate was rapidly formed. The precipitate was formed, cooled in an ice bath, and washed with water to remove traces of acid. The filtrate was dried under reduced pressure and characterized by means of ^1^H NMR.

#### 2.2.1 *C*-Tetramethylcalix[4]resorcinarene (Crown) **1a**

White solid in yield 37.5%. M.P. >250 °C decomposition. ^1^H NMR, δ (DMSO-*d*_6_, room temperature, ppm): CH_3_ (1.39, d), CH (4.45, q), aromatic CH (6.14, s), aromatic CH (6.77, s), OH (8.53, s).

#### 2.2.2 *C*-Tetramethylcalix[4]resorcinarene (Chair) **1b**

Cream solid in yield 6.25%. M.P. >250 °C decomposition. ^1^H NMR, δ (DMSO-*d*_6_, room temperature, ppm): CH_3_ (1.15, d), CH (4.37, q), aromatic CH (6.08, 6.17, s), aromatic CH (6.26, 6.79, s), OH (8.41, 8.65, s).

#### 2.2.3 *C*-Tetramethylcalix[4]resorcinarene (Diamond) **1c**

Cream solid in yield 3.6%. M.P. >250 °C decomposition. ^1^H NMR, δ (DMSO-*d*_6_, room temperature, ppm): CH_3_ (1.15, 1.40), CH (4.38–4.46), aromatic CH (6.12, 6.16, 6.21, 6.29), aromatic CH (6.86, 6.90, 6.98, 7.29), OH (8.50–8.90).

### 2.3 LC Q-TOF MS

All products were analyzed on a Bruker Impact IILC Q-TOF MS equipped with electrospray ionization (ESI) in positive mode. Chromatographic conditions were: Intensity Solo C18 column (2.1 × 100 mm, 1.8 μm) (Bruker Daltonik), at a temperature of 40 °C and a flow rate of 0.250 mL/min. Mobile phase water (A) and acetonitrile (B), each containing 0.1% formic acid. Gradient elution 5/5/95/95/5/5% B in 0/1/11/13/13.1/15 min. ESI source conditions: end plate offset 500 V, capillary 4500 V, nebulizer 1.8 bar, dry gas nitrogen 8.0 L/min, dry temperature 220 °C. Scan mode Auto MS/MS with spectral range 20–1000 *m*/*z*, spectra rate 2 Hz, and collision energy of 5.0 eV.

### 2.4 Separation of the Mixture by RP-HPLC

RP-HPLC analyses were performed on a Chromolith RP-18e (50×4.6 mm) using an Agilent 1200 liquid chromatograph (Agilent, Omaha, NE). A gradient elution of solvent B (TFA 0.05% in acetonitrile) in solvent A (TFA 0.05% in water) was performed as follows: 5/5/100/100/5/5% B at 0/1/18/21/21.1/24 min. Detection was performed at 210 nm, and the flow rate was 2 mL/min. The sample concentration of *C*-tetramethylcalix[4]resorcinarene (conformational mixture) was 1.0 mg/mL, and 10 μL was injected.

### 2.5 Separation of the Mixture via SPE

Supelclean ENVI-18 SPE cartridges (bed wt. 5 g, volume 20 mL) were used. SPE columns were activated prior to use with 30 mL of methanol and 30 mL of ACN (containing 0.1% TFA, solvent B) and were equilibrated with 30 mL of water (containing 0.1% TFA, solvent A). *C*-Tetramethylcalix[4]resorcinarene (conformational mixture) (46 mg) was dissolved in 1000 μL of ACN/H_2_O (50:50), and the solution was added to the column. The conformational mixture elution was performed by increasing the percentage of solvent B in the eluent. The collected fractions were analyzed via RP-HPLC. The fractions containing the pure conformer were mixed and then lyophilized.

## 3 Results and Discussion

**Figure 2:**
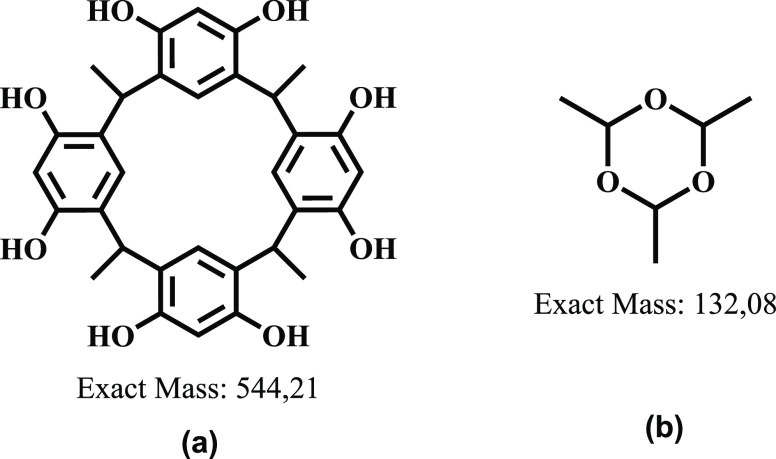
Possible products formed during the cyclocondensation reaction between acetaldehyde and resorcinol: (a) *C*-tetramethylcalix[4]resorcinarene conformers and (b) 2,4,6-trimethyl-1,3,5-trioxane.

As can be found in the literature,^22−24^ there are several synthesis procedures that can be carried out between resorcinol and acetaldehyde. These methods involve changing conditions such as pH and temperature, among others. Considering several of these published articles, we proposed to carry out a reaction between acetaldehyde and resorcinol using the procedure described by Hoegberg et al.^22^ As it is shown in the Material and Methods (section 2.2), the synthesis of resorcinarene was carried out through the acid-catalyzed cyclocondensation of resorcinol with acetaldehyde in water heated at reflux for 1 h. The solid product formed was filtered, washed, and dried in accordance with the conventional purification process. Preliminary TLC analysis of the solid showed the formation of two products; however, ^1^H NMR analysis of the solid showed several resonance signals for the aromatic hydrogen atoms for the conformer mixture at 6.08–6.79 ppm, the methylene bridges fragments at 4.37 and 4.45 ppm, and hydroxyl moieties (*d* = 8.41–8.65 ppm). Due to the complexity of the signals observed in the ^1^H NMR spectrum by means of the methodology adapted from the literature, this contrasts with the results obtained previously,^22,24^ in which it was reported that the cyclocondensation reactions allow the formation of two conformers (chair and crown). The formation of a third product was seen, as evidenced by the number of signals in the aliphatic region, which can be considered to be a third conformed, or the formation of trioxane ring (Figure 2), which can be formed even under the working reaction conditions.^25,26^

**Figure 3:**
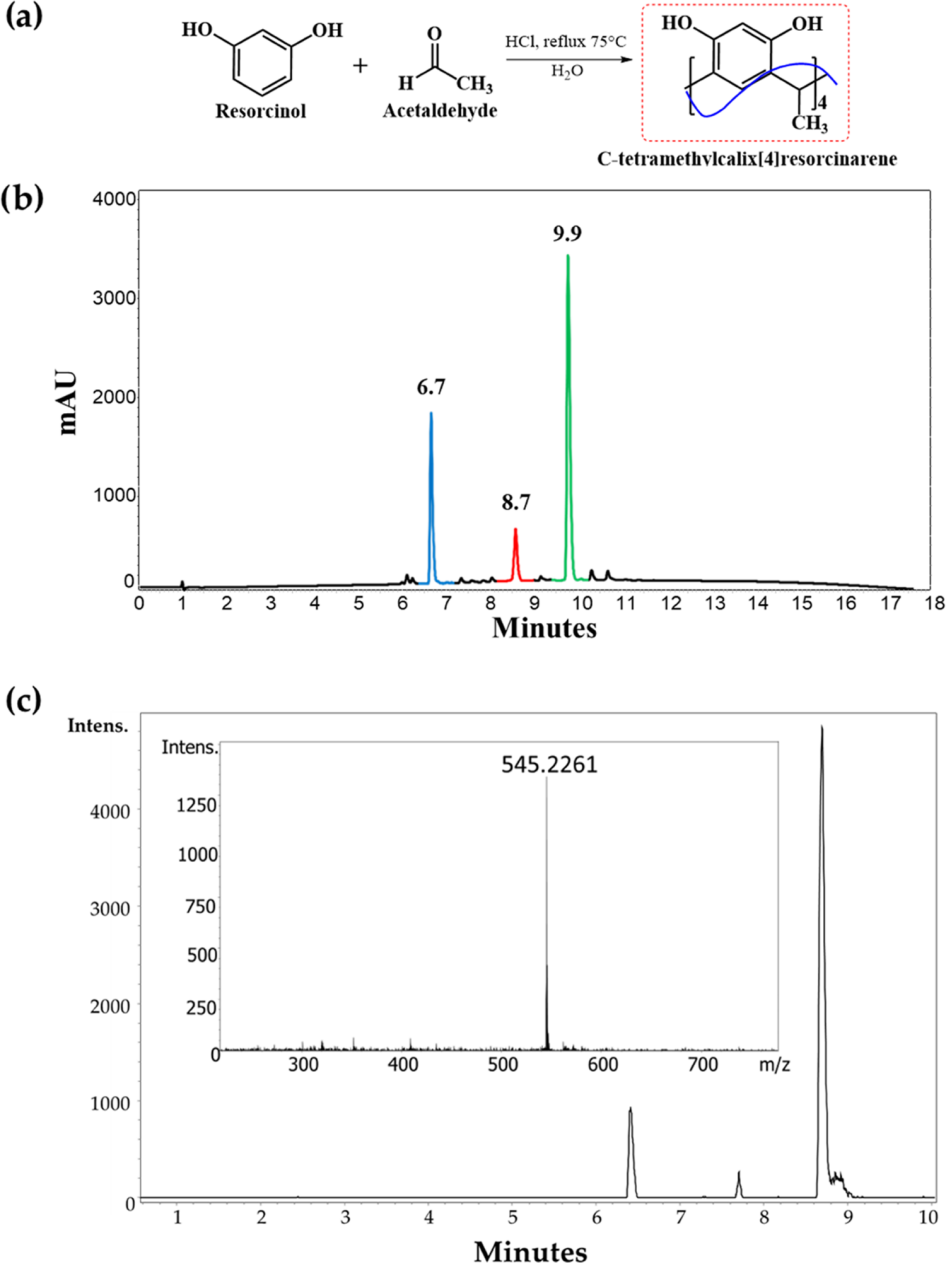
(a) Reaction of cycloaddition between resorcinol and acetaldehyde. Reaction mixture analysis by (b) RP-HPLC/UV–Vis, analysis recorded at 210 nm, and (c) UPLC-MS (ESI). TIC and MS spectra of the peak at 8.7 min.

As mentioned, these results were interesting since the characterization of three products from the cyclocondensation between acetaldehyde and resorcinol was not found in the consulted literature. To establish if the reaction mixture corresponded to three structural isomers or perhaps one of the products corresponded to a trioxane-type intermediate, we proposed to establish which of the two product types could be formed. Therefore, first, the mixture was analyzed by means of RP-HPLC with a UV/vis detector (210 nm), and then two C18 columns were tested, a packet and a monolithic. The optimized separation method (using the monolithic column) showed a chromatographic profile in the presence of three well-resolved peaks at *t*_R_ = 6.7, 8.7, and 9.9 min (Figure 3b). Then, the reaction mixture was analyzed via UPLC and electrospray ionization (ESI)-mass spectrometry (MS), recording in the positive-ion mode. The chromatographic profile also showed three principal peaks, and all of them exhibited an MS spectrum with a signal at *m*/*z* 545.21 corresponding to the [M + H]^+^ species (Figure 3c). According to the information obtained from the UPLC-MS analysis, the formation of three products was confirmed. Additionally, it can be deduced that the three products are isomers.

Once it was established that the solid product obtained corresponds to a mixture of three isomers, it was decided to separate them using the previously described RP-SPE technique.^20,21^ From the RP-HPLC profile, using eq 1, the percentage of solvent B (TFA 0.05% in acetonitrile) was calculated, in which each conformer eluted (%*B*_e_).
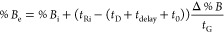
1Specifically, the RP-HPLC used elution program was: 5/5/100/100/5/5% B at 0/1/18/21/21.1/24 min, where *t*_G_ = 17 min, *t*_delay_ = 1 min, %*B*_i_= 5% and Δ%*B* = 95%. Additionally, the HPLC dwell time (*t*_D_) and column dead time (*t*_0_) were previously measured and they correspond to 0.9 and 0.82 min. As an example, eq 2 shows the determination of %*B*_e_ for a peak at 6.7 min.
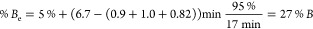
2Equation 1 allowed us to establish that the peak at retention times of 6.7, 8.7, and 9.9 min eluted at 27, 38, and 45% of solvent B, respectively. This information allowed us to design a gradient elution program for separating the conformers by means of the SPE technique. The planned program initiated with a 5% B fraction and increased then to 10, 15, 17, 19, 22, 24, 26, 28, 30, 33, 35, 36, 37, 40, 42, 50, and 100% B; 10 mL of each fraction were prepared.

Then, 45.6 mg of the conformational mixture was dissolved in 1000 μL of ACN/H_2_O (50:50) and loaded onto a 5 g RP-SPE cartridge. The elution was performed by increasing the percentage of solvent B in the eluent. The collected fractions were analyzed online, via UV–vis dispositive, and then by RP-HPLC, to determine the chromatographic purity. The compound corresponding to the peak with *t*_R_ = 6.7 min (Figure 3b) eluted in a range of solvent B of 19–26% B, the second compound (*t*_R_ = 8.7 min, Figure 3b) eluted in a range of 33–35% B, and finally the majority species, (*t*_R_ = 9.9 min) eluted in a %*B* range of 37–42. The fractions containing each pure conformer were mixed and then lyophilized. This method furnishes products with great purity; there is no need for sophisticated equipment, and the consumption of the mobile phase is minimal.

**Figure 4:**
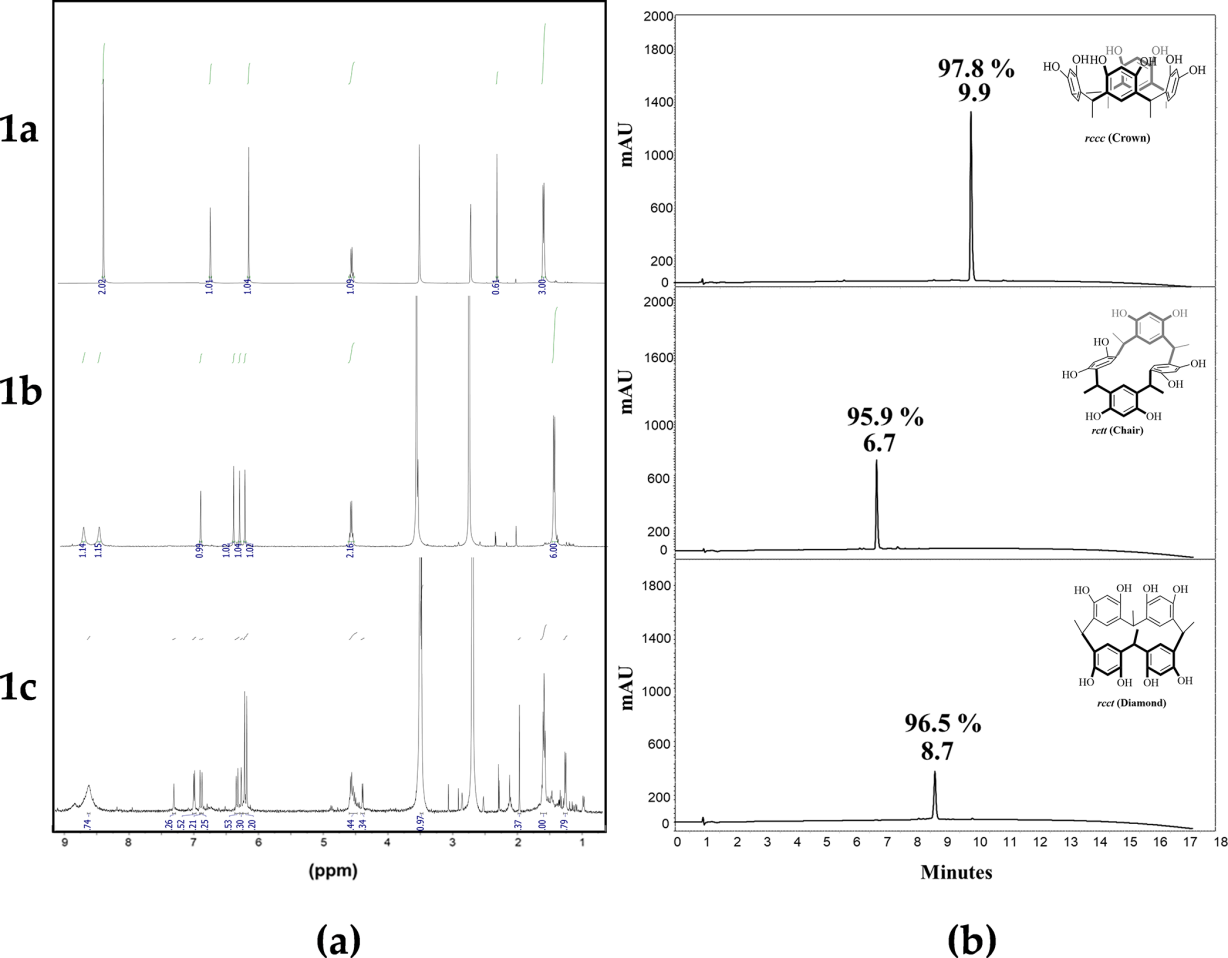
(a) ^1^H NMR spectra and (b) chromatographic profile of **1a**, **1b**, and **1c**; for each peak, the chromatographic purity (%) obtained after the RP-SPE process is shown.

The purified products were quantified, and the conformations of the macrocycle derivatives were established via ^1^H NMR spectroscopy in DMSO-*d*_6_ at room temperature (Figure 4). The ^1^H NMR spectra of the majority product (*t*_R_= 9.9 min) showed a signal at 8.53 ppm assigned to hydroxyl groups attached to resorcinol residues in the macrocyclic system. In the aromatic region, two signals can be seen in the resorcinol residues, one corresponding to the protons in the ortho position with respect to the hydroxyl group at 6.15 ppm and the other signal corresponding to the protons in the meta position with respect to the hydroxyl groups at 6.77 ppm. In the aliphatic region, the compound displayed the characteristic signal of a methine bridge at 4.45 ppm and the signal at 1.39 ppm corresponding to the methyl group. So, all of the patterns were consistent with the structure of the expected crown conformer **1a**, which has fewer signals in the spectrum.^24^ On the other hand, the ^1^H NMR spectra of product **1b** (*t*_R_ = 6.7 min) showed two signals, at 8.41 and 8.65 ppm, corresponding to two classes of hydroxyl groups attached to resorcinol residues in the macrocyclic system. In the aromatic region, four signals can be seen in the resorcinol residues, two corresponding to the protons in the ortho position with respect to the hydroxyl group at 6.08 and 6.17 ppm and the other two corresponding to the protons in the meta position with respect to the hydroxyl groups at 6.26 and 6.79 ppm. In the aliphatic region, two signals were observed, at 4.37 and 1.15 ppm, corresponding to the methine and methyl groups. Thus, these patterns were consistent with the structure of the expected chair conformer.^24^

Product **1c** (*t*_R_ = 8.7 min) showed a strong tendency for decomposition; for this reason, its characterization was only carried out in solution. As shown in Figure 4, the complete absence of symmetry in conformer **1c** is reflected in the ^1^H NMR spectra, so the number of signals for the product contrasts with the number of signals of crown and chair conformers. The ^1^H NMR spectrum of **1c** displayed the characteristic signals of a methyl substituent at 1.35 and 1.40 ppm and in the range of 4.38–4.46 ppm for the methine bridge. The aromatic protons at 6.12, 6.16, 6.21, and 6.29 ppm were assigned to the protons in the ortho position of hydroxyl groups for the resorcinol residue. Also, in the aromatic region, signals for meta-protons of the resorcinarene moiety were observed at 6.86, 6.90, 6.98, and 7.29 ppm. The signal for the hydroxyl groups was observed in the range of 8.50–8.90 ppm; the full assignment can be seen in Table 1. Finally, the integration of the signals is consistent with a tetrameric macrocycle. The multiplicity of signals for each type of proton in the spectrum of **1c** suggests different chemical environments for each of the phenolic rings, so the spectral pattern is characteristic of the diamond conformation.

**Table 1:**
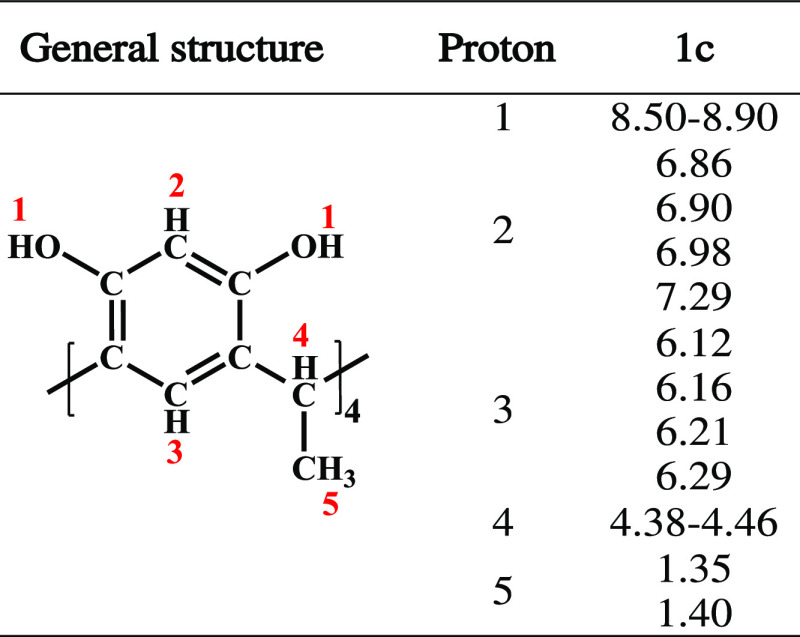
Chemical Shifts for Isomer **1c** in the ^1^H NMR Spectrum

**Figure 5:**
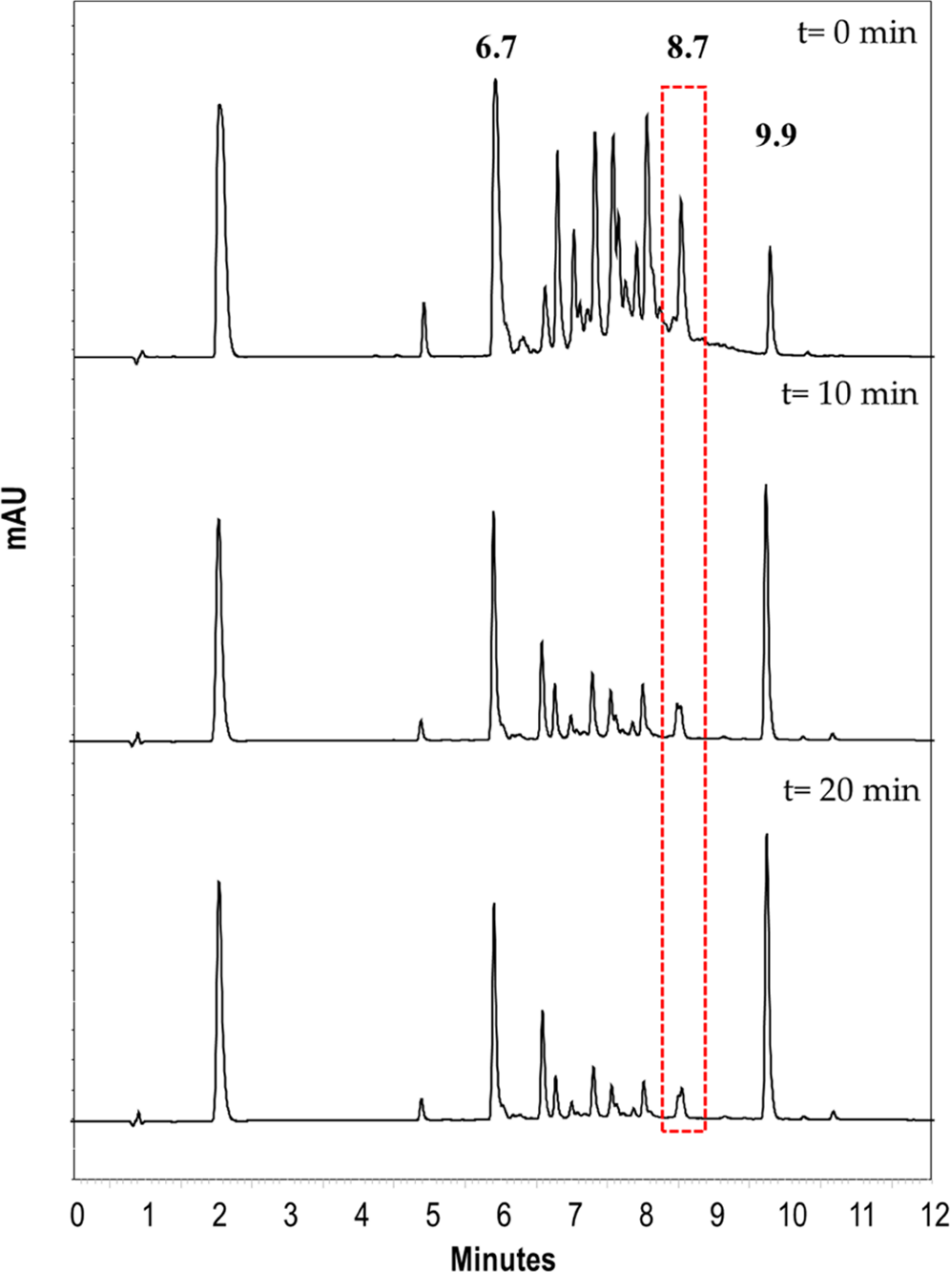
Chromatographic profile of the cyclocondensation reaction between resorcinol and acetaldehyde over time.

To understand the formation of **1a** during the cyclocondensation reaction between resorcinol and acetaldehyde, the chromatographic profile of the reaction solution was obtained from time 0 to 60 min (reaction time), obtaining the chromatogram every 10 min. As shown in Figure 5, during the first moments of the reaction, conformer **1c** is formed in good proportion; however, as the reaction progresses, the amount that is formed tends to decrease. Considering that the most stable conformer is the crown, in which both OH groups of the two opposite resorcinol units oriented toward the cavity act as hydrogen-bond donors, conformers **1b** and **1c** can be interconverted to the more stable conformer **1a** since the energy values are relatively low.^27^ The composition of the reaction products reflects the balance between the rate of the conformational and the annulus inversion in the reaction conditions.

## 4 Conclusions

Analysis by means of RP-HPLC and LCMS (positive-ESI) of the cyclocondensation of resorcinol with acetaldehyde in water showed the formation of three of the several possible *C*-tetramethylcalix[4]resorcinarene conformers. With the RP-HPLC information, it was possible to design a purification protocol using RP-SPE and gradient elution. The purified products were well characterized, i.e., conformational analysis of all of these compounds was done via NMR. The crown conformation was the main product obtained, but the chair and diamond conformers were also generated with acceptable yields. The method developed was applied, and it has the advantages of high sensitivity, low running cost, and simple operation. The analytical separation process used can be adapted to carry out studies on the formation of conformers of analogous systems.
